# Nanoparticle contrast-enhanced computed tomography of sporadic aortic aneurysm and dissection: Effect of nanoparticle size and contrast agent dose

**DOI:** 10.7150/thno.109325

**Published:** 2025-02-24

**Authors:** Laxman Devkota, Chen Zhang, Deborah Vela, Poonam Sarkar, Prajwal Bhandari, Zbigniew Starosolski, Renuka Menon, Dianna M. Milewicz, Ying H. Shen, Scott A. LeMaire, Ketan B. Ghaghada

**Affiliations:** 1 Department of Radiology, Baylor College of Medicine, Houston, TX.; 2 Edward B. Singleton Department of Radiology, Texas Children's Hospital, Houston, TX.; 3 Division of Cardiothoracic Surgery, Michael E. DeBakey Department of Surgery, Baylor College of Medicine, Houston, TX.; 4 Department of Cardiovascular Pathology Research, Texas Heart Institute, Houston, TX.; 5 Department of Pediatrics, Baylor College of Medicine, Houston, TX.; 6 Department of Internal Medicine, University of Texas Health Science Center at Houston, Houston, TX.; 7 Department of Cardiovascular Surgery, Texas Heart Institute, Houston, TX.; 8 Cardiovascular Research Institute, Baylor College of Medicine Houston, TX.; 9 Research Institute and Heart & Vascular Institute, Geisinger, Danville, PA.

**Keywords:** Aortic dissection, aortic aneurysm, computed tomography, imaging, nanoparticle, aorta

## Abstract

**Background**: Aortopathies, such as aortic aneurysm and dissection (AAD), are associated with enhanced aortic wall permeability and endothelial dysfunction. We previously demonstrated that nanoparticle contrast-enhanced computed tomography (nCECT), which detects enhanced aortic wall permeability, could enable non-invasive detection of early AAD before its progresses to fatal aortic rupture. This study investigated the effect of nanoparticle contrast agent (NPCA) size and dose on detection of aortopathy by nCECT.

**Methods:** *In vivo* studies were performed in a mouse model of sporadic AAD induced by challenging animals with high fat diet (5 weeks) and angiotensin II infusion (last one week). The effects of NPCA size (80, 150, and 240 nm) and NPCA dose (300, 600, and 1200 mg I/kg) on detection of aortopathy were studied. To examine temporal changes in aortic wall NPCA signal at sites of AAD, mice underwent longitudinal CT. To investigate changes in aortic wall integrity, mice underwent follow-up nCECT at 6 months after initial challenge. Imaging findings were compared with gross and histologic examination of the aorta. Fluorescence microscopy was used to confirm presence or absence of intramural NPCA.

**Results**: nCECT using all three sizes of NPCAs demonstrated significantly higher sensitivity (p< 0.05) for the detection of aortopathy compared to gross examination. Histologic analysis showed excellent correlation between the nCECT finding of intramural signal and the presence of aortopathy. The absolute improvements in detection rates were 16%, 20%, and 17% for 80 nm, 150 nm, 250 nm NPCA respectively. Sensitivity of nCECT for detection of aortic injury improved with increasing NPCA dose compared to gross exam (-17% at 300 mg I/kg to 14% at 1200 mg I/kg). Temporal analysis of aortic wall NPCA signal at sites of AAD demonstrated a peak in aortic wall CT attenuation at day 3-5 post-contrast followed by gradual return to baseline by day 120. Follow-up nCECT at 6 months demonstrated absence of wall signal enhancement compared to baseline, suggesting resolution of the altered aortic wall permeability and injury. Histologic analysis demonstrated remodeling and healing of the aortic wall.

**Conclusions**: Nanoparticle contrast-enhanced CT using all three studied nanoparticle sizes demonstrated higher sensitivity than gross examination for the detection of aortopathy. A dose-dependent effect on sensitivity was observed with only high NPCA dose (1200 mg I/kg) demonstrating superior performance compared to gross examination for detecting early stages of aortic injury. Nanoparticle contrast-enhanced CT enabled *in vivo* interrogation of changes in aortic wall integrity.

## Introduction

Aortic aneurysm and dissection (AAD) are characterized by progressive weakening, dilatation and tearing of layers within the aortic wall 1-3. These common interrelated cardiovascular pathologies progress to lethal aortic rupture claiming over 10,000 lives in the United States every year 1,3,4. AAD is generally diagnosed by evaluating aortic diameter on cross-sectional imaging, such as computed tomography angiography (CTA) or magnetic resonance angiography (MRA). Although these imaging techniques exhibit high sensitivity for detection of aortic enlargement, they are unable to detect microstructural changes occurring in the early stage of pathogenesis that precedes aneurysmal dilatation or dissection 5-9.

Unlike conventional small molecule contrast agents, the unique physico-chemical properties of a nanoparticle contrast agent (NPCA), namely large size and long blood half-life, provide distinct advantages for disease interrogation 10-13. In a mouse model of sporadic AAD, we have previously shown that nanoparticle contrast-enhanced computed tomography (nCECT) using a long-circulating liposomal-iodine NPCA enabled early detection and *in vivo* imaging of AAD due to aortic wall signal enhancement at sites of aortopathy 14. nCECT demonstrated higher sensitivity for *in vivo* detection of microstructural wall degeneration compared to CTA and gross examination of the aorta 14. Mechanistic studies revealed increased aortic permeability associated with intimal and medial tears as the driving factor for intramural accumulation of NPCA and higher sensitivity of nCECT.

In this preclinical study, we investigated the effect of NPCA size and NPCA dose on the performance of nCECT for detection of aortopathy. Furthermore, longitudinal CT was performed to study *in vivo* temporal changes in aortic wall NPCA signal at sites of aortopathy and to interrogate changes in aortic wall integrity.

## Materials and Methods

### Nanoparticle Contrast Agent (NPCA)

Liposomal-iodine NPCAs of three sizes were fabricated and characterized as per methods described previously 15-18. Briefly, a lipid mixture consisting of L-α-phosphatidylcholine, hydrogenated (Hydro Soy PC; HSPC), cholesterol, 1,2-distearoyl-sn- glycero-3-phosphoethanolamine-N-[methoxy(polyethylene glycol)-2000] (DSPE-mPEG2000; Lipoid GmbH, Ludwigshafen, Germany), and rhodamine B 1,2-dihexadecanoyl-sn-glycero-3-phosphoethanolamine (Thermo Fisher Scientific, Waltham, MA) were dissolved in ethanol at a molar ratio of 54.8:40:5:0.2. The ethanolic lipid solution was hydrated with an iodixanol solution (525 mg I/mL; Hovione, Loures, Portugal) at 60-65ºC and sequentially passed through a high-pressure extruder containing filters with a defined pore size (400, 200 and 100 nm) to generate liposomes of different mean sizes (240, 150 and 80 nm). The resulting solution was diafiltered against 150 mM NaCl/10 mM histidine to remove unencapsulated iodixanol.

Size analysis of NPCA formulations was performed using a dynamic light scattering instrument. The iodine content of NPCA was determined using UV spectrophotometry. Phospholipid content was determined by measuring phosphorus using inductively coupled plasma atomic emission spectroscopy (ICP-AES). The stability of NPCAs was determined using an *in vitro* release assay performed at 37ºC using bovine plasma.

### *In vivo* studies

Animal studies were performed under a protocol approved by the Institutional Animal Care and Use Committee.

#### Animal model

*In vivo* studies were performed in a mouse model of sporadic AAD 19-21. Four-week-old male and female C57BL/6J mice (The Jackson Laboratory, Bar Harbor, ME, and Gnotobiotics Core, Center for Comparative Medicine, Baylor College of Medicine, Houston, TX) were challenged with high-fat diet (HFD; 20% protein, 40% carbohydrate, 40% fat, and 1.25% cholesterol; Research Diets Inc, New Brunswick, NJ) and angiotensin II (Ang II; 2000 ng/min per kg; Sigma Aldrich Corp, St. Louis, MO) delivered using a subcutaneous osmotic minipump (ALZA Scientific Products, Mountain View, CA).

#### Effect of NPCA size

Thirty-six challenged mice (HFD + Ang II) were randomized to one of three NPCA size groups (n=12/group, 6F+6M): 80, 150 and 240 nm. Mice were intravenously administered NPCA (dose: 1200 mg I/kg) one day after starting Ang II infusion. Animals underwent nCECT at 4 days post-contrast. Immediately thereafter, a second dose (1200 mg I/kg) of NPCA (without rhodamine B) was administered and CTA performed to opacify the aortic lumen and aid with *in vivo* localization of signal enhancement. Animals were euthanized immediately after CTA and aortas excised for *ex vivo* nCECT and microscopic analysis. Due to the sporadic nature of AAD development in this model, animals can die prematurely due to aortic rupture. Additional animals were recruited into the study if animals died prematurely due to aortic rupture.

#### Effect of NPCA dose

Thirty-six challenged mice (HFD + Ang II) were randomized to one of three NPCA dose groups (n=11/group, 5F+6M): 300, 600 nm and 1200 mg I/kg. Mice were intravenously administered NPCA (150 nm size) one day after starting Ang II infusion. Animals underwent nCECT at 4 days post-contrast. Immediately thereafter, CTA (1200 mg I/kg, 150 nm) was performed to opacify the aortic lumen and aid with *in vivo* localization of signal enhancement. Animals were euthanized immediately after CTA and aortas excised for *ex vivo* nCECT and microscopic analysis.

#### Longitudinal monitoring of aortic wall signal

To examine temporal changes in aortic wall NPCA signal at sites of AAD, animals underwent longitudinal CT imaging. Challenged male mice (n=15) underwent pre-contrast baseline CT followed by intravenous NPCA administration (1200 mg I/kg, 150 nm size) one day after starting Ang II infusion. Angiotensin infusion was stopped after one week, and animals were placed on regular diet for the remainder of the experiment. Longitudinal CT was performed post-contrast at 3, 5, 28, 120, and 180 days. A subset of mice (n=6) died during the study due to aortic rupture; remaining mice (n=9) survived until the end of study.

#### Interrogation of aortic wall permeability

To investigate utility of nCECT for probing changes in aortic wall permeability, animals from the above study underwent follow-up nCECT at 6 months after initial challenge. Animals were intravenously administered NPCA (1200 mg I/kg, 150 nm size) and immediately underwent CTA. Subsequently, animals underwent nCECT at 4 days post-contrast. Animals were euthanized after nCECT and aortas harvested for microscopic analysis.

### CT imaging

Imaging was performed on a small animal micro-CT scanner (Inveon, Siemens, Inc, Knoxville, TN) 19. Mice were placed in prone position on CT scanner bed and imaged while free breathing under anesthesia (1.5%-2.5% isoflurane) delivered using a face cone setup. The respiratory rate was monitored using a pressure pad placed under the mouse's abdomen. CT images were acquired using the following scan parameters: 50 kVp peak voltage, 0.5 mA tube current, 400 ms x-ray exposure time, 540 projections. Scan time was 10 minutes and the estimated radiation dose, determined using a point source dosimeter, was 30 cGy. Images were reconstructed using filtered back-projection at an isotropic resolution of 70 μm. CT images were calibrated for Hounsfield unit (HU). Whole-body CT images were acquired to capture all segments of the aorta.

### Postmortem analysis of aorta

Animals were euthanized after final imaging session. Aortas were carefully excised under a dissecting microscope and perfused with saline solution to eliminate potential CT signal confounders like pooled blood or residual contrast agent in the lumen and then fixed in formalin 14,19,21.

#### Gross examination

Gross examination of excised aorta was performed by one grader, blinded to study groups, to evaluate severity of AAD according to a scheme based on the classification system described previously: preclinical (no gross findings but disease findings in nCECT), mild disease (dilation), and advanced disease (dilation with signs of intramural hemorrhage/dissection or rupture) 14,19. The incidence of aortic findings was determined for three segments of the aorta: ascending aorta and arch, descending thoracic aorta (DTA), and suprarenal abdominal aorta (SRAA).

#### *Ex vivo* nCECT

To confirm *in vivo* nCECT findings of wall signal enhancement, *ex vivo* nCECT was performed on excised aortas using CT scan protocol described above.

#### Histologic evaluation

After gross examination and *ex vivo* CT, formalin-fixed aortas were transferred into optimal cutting temperature (OCT) medium and stored frozen at -80°C. nCECT and gross examination findings were used to identify and select sites of peak injury within each respective aortic segment for sampling. Adjacent cryosections were cut at 5 µm thickness and were stained with hematoxylin and eosin (H&E) and Movat pentachrome. Additional sections were collected for immunofluorescence analysis (described below). Microphotographic images were captured at 4x, 10x, and 20x magnification using an Olympus BX61 microscope for histologic evaluation. Aortic wall lesions were semi-quantitatively assessed by a cardiovascular pathologist, blinded to study groups, using the following parameters: presence and location of dissections, intralaminar hematoma; location and count of intimal-medial tears; grade, extent and location of interlaminar space expansion; location and distribution of interlaminar ECM deposition; location and pattern of inflammation.

#### Intramural nanoparticle accumulation and immunofluorescence analysis

Intramural presence of rhodamine-labeled NPCA in aortic sections was analyzed in the red channel (emission/excitation, 590±25/568 nm). Cryosections were stained with anti-CD68 antibody (Abcam, Cambridge, United Kingdom; ab125212; 1:200 dilution with PBS+1% BSA) to evaluate presence of macrophages and active inflammation as described previously 19,22. Briefly, frozen aortic segments embedded in OCT (Sakura Finetek, Torrance, CA) were cut into 5-mm sections. Optimal cutting temperature-embedded aortic sections were fixed with Cytofix (BD Biosciences, San Jose, CA) and permeabilized with Perm/Wash (BD Biosciences). Nonspecific staining was reduced by blocking with 10% donkey serum. Sections were then incubated with primary antibody at room temperature for 2 hours or at 4°C overnight, washed with PBS, and incubated with fluorophore-labeled secondary antibody (Invitrogen, Waltham, MA; A-11008; 1:500 dilution with PBS+1% BSA) at room temperature for 1 hour). Nuclei were counterstained with 4′,6-diamidino-2-phenylindole. The slides were mounted with Dako Fluorescence Mounting Medium (Dako North America, Inc, Carpinteria, CA) and analyzed with fluorescence microscopy. All sections were examined using a Leica SP5 confocal microscope (Leica Microsystems, Inc, Wetzlar, Germany) or an Olympus immunofluorescence microscope (Tokyo, Japan). Exposure time was kept constant for all examined tissue sections.

### CT image analysis

CT images were analyzed using Osirix (version 5.8.5, 64-bit; Pixmeo SARL, Geneva, Switzerland) and MATLAB (version 2019a; MathWorks, Natick, MA) as per methods described previously 14.

#### Analysis of aortic wall signal enhancement

For NPCA size and dose study, the incidence of aortic findings (defined as wall signal enhancement) in *in vivo* nCECT and *ex vivo* nCECT images was determined by review of cross-sectional images. Pseudo-colored *ex vivo* nCECT images were generated to aid in the visualization of wall signal enhancement. Analysis was performed in three segments of the aorta: ascending aorta and arch, descending thoracic aorta (DTA), and suprarenal abdominal aorta (SRAA). For longitudinal monitoring of aortic wall signal, quantitative measurement was performed in three segments of aorta described above. Three dimensional ROIs were manually drawn around aortic wall regions showing signal enhancement on nCECT images. Segmented volumes were thresholded to separate signal-enhancing pixels from pixels with background signal using methods described previously 14,23. Each selected region was processed locally to prevent overestimation of threshold value by using the iterative selection method implemented in ImageJ. Analysis was performed on pre-contrast baseline images and post-contrast nCECT images.

### Data and statistical analysis

The nonparametric paired Wilcoxon signed-rank test was performed using the statistical toolbox of MATLAB software to evaluate differences in incidence of aortic findings determined using nCECT and gross examination. Incidence of aortic findings in *in vivo* nCECT images and gross observation of aortic pathology was determined by counting aortic segments that showed signal enhancement. For long-term monitoring of intramural aortic wall signal, Wilcoxon matched pairs signed rank test was used to analyze differences in aortic wall signal at different time points. Bonferroni correction was applied for multiple group comparisons.

## Results

### NPCA formulations

Iodine-encapsulated liposomal NPCA formulations were fabricated at three different sizes. Particle size analysis demonstrated an average hydrodynamic diameter of 85 ± 3 nm (referred as *80 nm NPCA*), 147 ± 8 nm (referred as *150 nm NPCA*), and 239 ± 12 nm (referred as *240 nm NPCA*) and a polydispersity index (PDI) of 0.09, 0.06, and 0.08, respectively. The iodine content was 73 mg I/mL for 80 nm NPCA, 87 mg I/mL for 150 nm NPCA, and 104 mg I/mL for 240 nm NPCA. *In vitro* release assay conducted in bovine plasma at 37ºC demonstrated less than 1% iodine leak for all NPCA formulations.

### Effect of NPCA size on detection of aortic dissection

**Figure 1:**
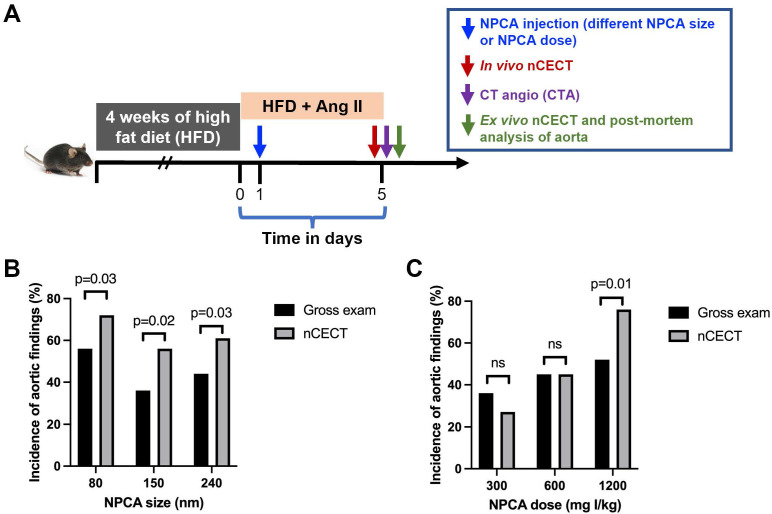
** Effect of NPCA size and dose on detection of aortopathy. (A)** Experimental design for *in vivo* testing of NPCA size and dose in challenged mice. HFD: high fat diet; Ang II: angiotensin II. **(B)** Incidence of aortic findings as a function of NPCA size. **(C)** Incidence of aortic findings as a function of NPCA dose. Aortic findings were determined from gross examination of excised aorta and presence of aortic wall signal enhancement in nCECT. 10-12 mice (24 - 36 aortic segments) per group were included in the analysis. Statistical analysis was performed using non-parametric paired Wilcoxon signed-rank test.

To study the effect of NPCA size on detection and imaging of aortic degeneration in the sporadic mouse model of AAD, challenged mice were intravenously administered one of three liposomal NPCAs (80 nm, 150 nm, or 240 nm size NPCAs) **(Figure 1A)**. The incidence of aortic findings (defined as wall signal enhancement) on nCECT was compared to pathological findings on gross and microscopic examination of excised aorta. Histological analysis of aortic tissue sections confirmed dissection as evidenced by intimal-medial tear and/or presence of intramural hematoma. *In vivo* nCECT and *ex vivo* nCECT of excised aorta demonstrated 100% concordance of aortic findings. nCECT using NPCAs of all sizes demonstrated significantly (p < 0.05) higher incidence of aortic findings compared to gross examination findings of aortic pathology (**Figure 1B**). The absolute improvements in detection rates of aortic injury using nCECT in comparison to gross observation were 16%, 20%, and 17% for 80 nm, 150 nm, and 240 nm NPCA respectively.

**Figure 2:**
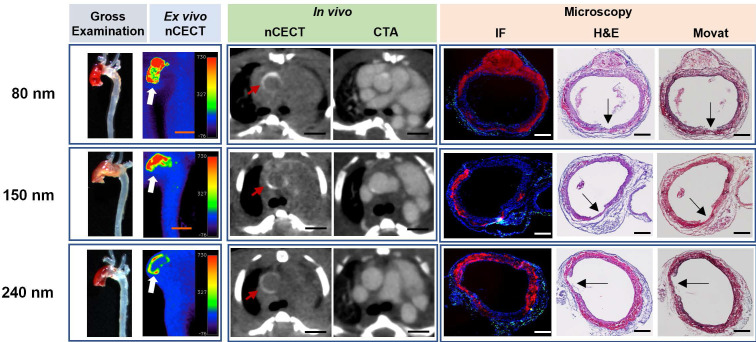
** nCECT of advanced aortopathy using different sizes of NPCA.** Representative examples of images acquired using 80nm, 150 nm, and 250 nm size NPCAs in mice with advanced aortopathy. Gross examination images demonstrate aortic pathology in ascending and arch segments. *Ex vivo* pseudo-colored nCECT maximum intensity projection (MIP) images demonstrate signal enhancement in aortic regions that show pathology on gross exam. *In vivo* axial nCECT images acquired 4 days post-contrast demonstrate wall signal enhancement (red arrow) with all three NPCA sizes; CTA confirmed association of wall enhancement in thoracic segment of aorta. Immunofluorescence (IF) confirmed intramural accumulation of rhodamine-labeled NPCA (red signal) for all particle sizes. Presence of wall inflammation at sites of aortic pathology was examined by staining for CD68^+^ macrophages (green signal). Histologic analysis (H&E and Movat staining) demonstrated a dissection in each case, as evidenced by a focal intimal-medial tear (arrows), intralaminar hemorrhage and expansion, and adventitial inflammation. Scale bar indicates 2.5 mm for nCECT images, and 200 µm for microscopic images.

All three sizes of NPCAs (80 nm, 150 nm, and 240 nm) demonstrated large regions of wall signal enhancement in aortas that showed moderate to severe aortic pathology on gross examination (**Figure 2**). Histological analysis with H&E and Movat staining confirmed moderate to severe aortic wall injury sites, most often corresponding to dissections with moderate to severe medial tears, and interlaminar hemorrhage and space expansion ranging from 60 to 100% of the wall circumference. Fluorescence microscopy confirmed intramural accumulation of liposomal nanoparticles for all three sizes of NPCAs. NPCAs of all three sizes were detected beyond the intimal layer on microscopy and colocalized with the intramural hemorrhage, suggesting the ability for even large size NPCAs (240 nm) to penetrate deep within the aortic wall. Immunofluorescence CD68 antibody staining revealed different intramural patterns of macrophage and nanoparticle distribution with inflammation seen predominantly in adventitial layer. This observation is consistent with our previous findings and indicates loss of endothelial barrier and medial tears as the route for passive entry of NPCAs into the aortic wall.

**Figure 3:**
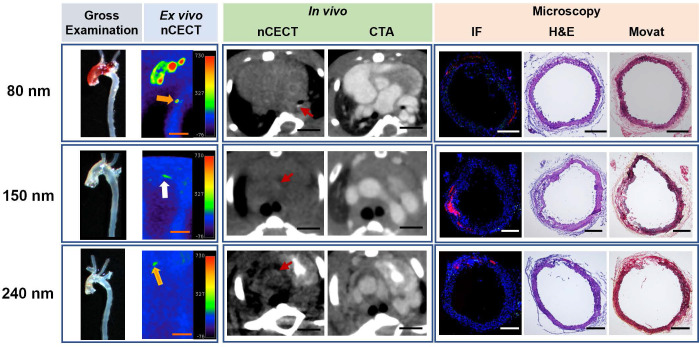
** nCECT of preclinical to mild aortopathy using different sizes of NPCA.** Representative examples of images acquired using NPCAs of different sizes in mice exhibiting preclinical to mild aortic pathology (in descending thoracic aorta in 80 nm; in arch in 150 nm; in ascending aorta in 240 nm). Gross examination and *ex vivo* nCECT images demonstrate preclinical disease (white arrow; not seen on gross exam images but show signal enhancement on nCECT) and mild disease (orange arrow; seen on gross exam and show signal enhancement on nCECT). *In vivo* axial nCECT images acquired 4 days post-contrast demonstrate subtle wall signal enhancement observed with all three particle sizes; CTA confirmed wall enhancement in aortic segments. Immunofluorescence (IF) demonstrated heterogenous intramural distribution of rhodamine-labeled NPCA (red signal) and mild inflammation (CD68+ macrophages, green signal) at aortic sites exhibiting mild disease. Histologic analysis (Movat and H&E staining) demonstrated less severe aortic injury as evidenced by mild inner medial tears, with less extensive or minimal interlaminar space expansion and negligible adventitial inflammation. Scale bars indicate 2.5 mm for nCECT images and 200 µm for microscopic images.

All three sizes of NPCAs (80 nm, 150 nm, and 240 nm) also demonstrated wall signal enhancement in aortas that showed preclinical (not visible on gross examination) or mild disease (seen on gross exam) (**Figure 3**). Region of wall signal enhancement on nCECT was smaller for mild AAD compared to advanced AAD. Despite subtle findings on gross examination, histological analysis confirmed aortic injury such as dissection sites showing mild or moderate medial tears and interlaminar hemorrhage and space expansion. Fluorescence microscopy showed mild and focal spots of fluorescence for all three sizes of NPCAs. Immunofluorescence staining with CD68 antibody revealed little to no intramural macrophages. In summary, nCECT using all three sizes of NPCAs demonstrated superior performance than gross examination for the detection of aortic dissections.

### Effect of NPCA dose on detection of aortic dissection

**Figure 4:**
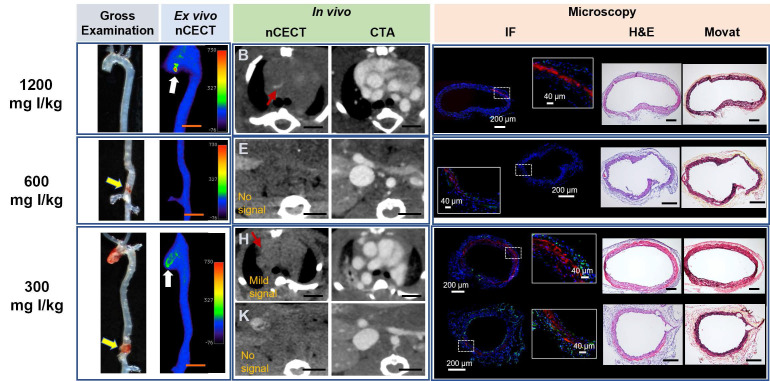
** Effect of NPCA dose on nCECT of aortopathy.** Representative images acquired in challenged mice administered 300, 600, and 1200 mg I/kg of NPCA dose. At 1200 mg I/kg NPCA dose, nCECT detects preclinical aortic disease (not seen on gross exam but show signal enhancement on nCECT; white arrow). An example at 600 mg I/kg dose shows aortic injury on gross examination (yellow arrows) but do not show signal enhancement in nCECT imaging. At 300 mg I/kg dose, aortic injury in the ascending segment shows nCECT signal; however, the lesion in the supra-renal abdominal aorta (SRAA) (yellow arrow) does not show nCECT signal. *In vivo* nCECT findings corroborate with *ex vivo* nCECT findings. Immunofluorescence (IF) demonstrate intramural NPCA accumulation (red signal) at 1200 mg I/kg dose. Minimal intramural NPCA accumulation (red signal) is seen in SRAA region for 600 mg I/kg NPCA dose. Interestingly, intramural NPCA accumulation (red signal) is seen at 300 mg I/kg in both regions of aortic injury, including in SRAA region that did not show signal enhancement on nCECT. CD68 staining (green signal) show mild diffuse inflammation in adventitia. Histologic analysis (Movat and H&E staining) demonstrates wide range of aortic injury in the form of mild inner medial tear, interlaminar hemorrhage and space expansion. Scale bars indicate 2.5 mm for nCECT images and 200 µm for microscopic images (40 µm for zoomed microscopic images).

To study the effect of NPCA dose on detection and imaging of aortic dissection in the sporadic mouse model of AAD, challenged mice were intravenously administered one of three liposomal NPCA doses (300, 600, or 1200 mg I/kg) **(Figure 1A)**. The incidence of aortic findings on nCECT was compared to pathological findings on gross examination of excised aorta. The absolute improvements in detection rates were -17% at 300 mg I/kg, no differences at 600 mg I/kg, and 14% at 1200 mg I/kg. Only at the highest dose (1200 mg I/kg), nCECT demonstrated superior performance (p< 0.05) than gross examination for the detection of aortic degeneration **(Figure 1C)**. At lower dose levels (300 and 600 mg I/kg), nCECT demonstrated similar or showed a trend of inferior performance compared to gross examination. In some cases, nCECT did not show signal enhancement at the site of aortopathy confirmed on gross examination; however, microscopic analysis demonstrated presence of fluorescently labeled NPCA **(Figure 4, 600 mg I/kg panel)**. This suggests that an adequate number of nanoparticles did not accumulate at the site of aortopathy to generate signal enhancement on nCECT. In another example **(Figure 4, 300 mg I/kg panel)**, nCECT demonstrated signal enhancement in one segment (ascending region) of the aorta but not in another region (suprarenal abdominal region), both of which demonstrated aortic injury in the form of focal intramural hemorrhage on gross examination. However, both injury sites demonstrated presence of fluorescently labeled NPCA on microscopic analysis. This suggests inherent variations in aortic wall permeability at different sites of aortic wall injury even within the same aorta. Furthermore, these findings suggest a specific threshold of iodine contrast needed for detection on CT.

### Temporal changes in aortic wall nCECT signal at sites of aortic dissection

**Figure 5:**
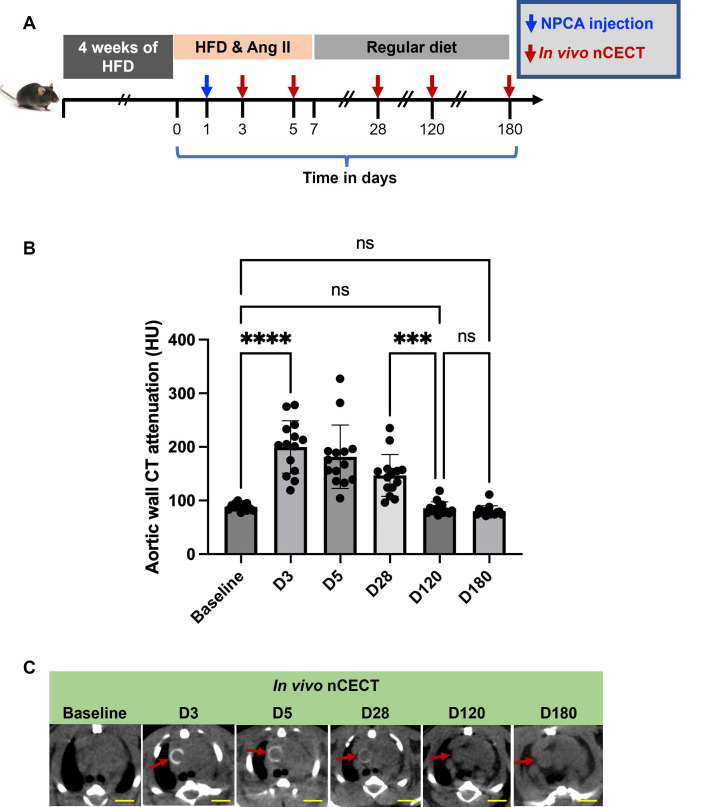
** Temporal changes in aortic wall signal at sites of aortopathy: (A)** Experimental design for studying temporal changes in aortic wall CT signal in challenged mice after single intravenous administration of NPCA. **(B)** Time-attenuation plot of aortic wall CT signal at baseline (pre-contrast) and at serial time points post-contrast. **(C)** Representative axial CT images showing temporal changes in wall signal (red arrow) in the ascending aortic segment of a challenged mouse. Scale bars = 2.5 mm for nCECT images. Statistical analysis was performed using Wilcoxon matched-pairs signed-rank test. Bonferroni correction was applied for multiple group comparison.

In a subset of challenged mice, longitudinal CT was performed after a single dose of NPCA to study temporal changes in aortic wall signal at sites of aortic degeneration **(Figure 5A)**. A peak in aortic wall CT attenuation was observed between day 3 and day 5 followed by gradual decay in aortic wall signal **(Figure 5B, C)**. Aortic wall CT attenuation returned to baseline by day 120.

### Interrogation of aortic wall integrity

**Figure 6:**
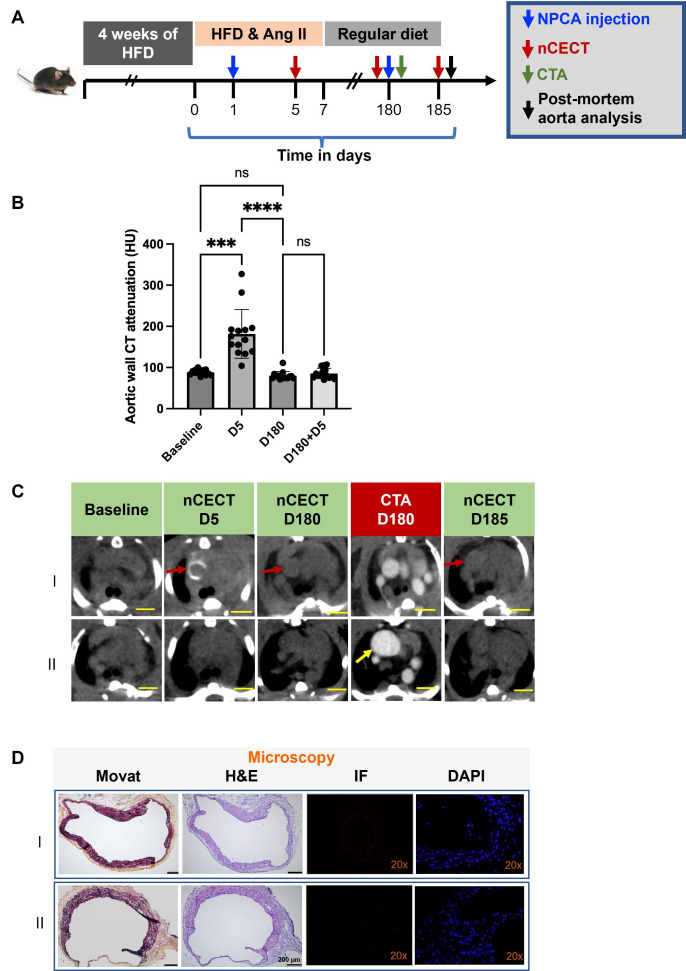
** Interrogation of aortic wall permeability using nCECT.** Experimental design to interrogate changes in aortic wall permeability in challenged mice. Mice were administered NPCA twice to probe changes in aortic permeability: at day 1 during the challenge period and at day 180 during recovery period. **(B)** Time-attenuation plot of aortic wall CT signal at baseline and after NPCA injections. Aortic wall signal is seen at day 5 during the challenge period due to aortic dissection. After a second NPCA injection at day 180, wall signal enhancement was not seen at day 185 suggesting restoration of aortic wall barrier. **(C)** Representative axial CT images showing changes in wall signal associated with changes in aortic wall integrity. Interestingly, a mouse with large aneurysm (bottom panel) did not show wall signal enhancement during challenge period or during recovery period, suggesting the presence of an intact aortic barrier throughout the duration of study. Scale bars indicate 2.5 mm for nCECT images.** (D)** Histology images show healed medial dissections displaying endothelialized medial flaps or short segments of the aortic wall consisting of only one or two outer medial laminar units and fibrotic adventitia (*arrows*). IF showed absence of labeled NPCA, consistent with nCECT at day 185, confirming no nanoparticle entry into the aortic wall. Scale bars indicate 2.5 mm for nCECT images and 200 µm for microscopic images (Movat and H&E). Magnification factor is 20x in IF and DAPI images. Statistical analysis was performed using Wilcoxon matched-pairs signed-rank test. Bonferroni correction was applied for multiple group comparison.

To investigate the utility of nCECT for interrogating the integrity of aortic wall, mice underwent a follow-up injection of NPCA at 180 days **(Figure 6A)**. CTA was acquired immediately after NPCA injection followed by nCECT 5 days later. nCECT did not show significant (p < 0.05) intramural signal enhancement at the same sites that had previously shown signal enhancement at day 5 **(Figure 6B, C)**. Given that the aortic lesions encountered in this model corresponded consistently with dissection sites, this suggests healing of these sites after discontinuation of Ang II and high fat diet challenge. Moreover, histopathology showed evidence of wall remodeling as evidenced by the presence of healed intimal-medial dissections displaying endothelialized medial flaps or short segments of the aortic wall consisting of only one or two outer medial laminar units and fibrotic adventitia **(Figure 6D)**. A resolved IMH was also observed in one mouse. Fluorescence microscopy demonstrated absence of labeled NPCA, thus providing further evidence of an intact aortic wall and endothelial barrier impeding entry and intramural accumulation of NPCA **(Figure 6D)**. One of the challenged mice did not show intramural wall CT signal enhancement after the first or second injection of NPCA despite exhibiting a large aneurysm in the ascending segment of the aorta, suggesting an intact intimal layer during the challenge period and throughout the remaining study duration **(Figure 6C-II)**.

## Discussion

Given the critical role of aortic wall permeability in the initiation and progression of aortic dissections, methods that enable *in vivo* detection of compromised aortic barrier integrity would help in advancing this field of vascular biology. In the current work, we investigated nCECT using different sizes of liposomal NPCAs and different doses of NPCA for the detection of aortopathy. Nanoparticle contrast agent of three different sizes (80, 150, and 240 nm mean particle size) were fabricated and tested in a mouse model of sporadic AAD. Regardless of NPCA particle size, nCECT imaging demonstrated higher sensitivity for the detection of aortic dissection compared to gross aortic examination. Furthermore, nCECT imaging using all NPCA sizes detected enhanced aortic wall permeability and intramural microstructural damage before pathological changes were evident on gross examination. These findings in this mouse model of sporadic AAD indicate that injury-associated morphological changes initiating in the intimal layer can facilitate intramural entry of large nanoparticles (240 nm as shown in this study).

The NPCA dose ranging study demonstrated that the sensitivity of nCECT for detection of aortic intramural injury improved with increasing NPCA dose. At lower dose level, nCECT missed detection of aortopathy, even though fluorescence microscopy revealed intramural accumulation of NPCA. This indicates poor intramural accumulation of nanoparticles at sites of aortopathy, resulting in signal enhancement below contrast threshold for CT detection. Interestingly, nCECT at low NPCA dose demonstrated heterogeneity in detection of aortopathy within the same animal. These finding highlights either differences in the size of the entry point (i.e., tear) and inherent intramural space for adequate nanoparticle accumulation at different dissection sites, or variability in the timing of the NPCA dose relative to the age of the injuries. While aortic degeneration associated with high aortic permeability can be detected with a lower NPCA dose (300 - 600 mg I/kg), aortic regions exhibiting mild-to-moderate lesions require a high NPCA dose (1200 mg I/kg). At 1200 mg I/kg, nCECT demonstrated superior performance than gross examination for the detection of aortopathy. Overall, these studies demonstrate that detection of aortopathy using nCECT depends on the size of the mural lesion, integrity of aortic wall barrier and NPCA dose.

The long-term study demonstrated the utility of *in vivo* nCECT for interrogating spatio-temporal changes in aortic wall permeability. Aortic regions that showed intramural CT attenuation during the Ang II and high fat diet challenge exhibited no signal enhancement at 6 months after the end of the challenge, suggesting healing of any prior injury site. Such methods could be helpful to non-invasively monitor therapeutic approaches targeting endothelial dysfunction-associated with aortic degeneration.

In this study, we compared nCECT findings to gross aortic examination findings and confirmed them with histological findings. Both, when combined, served as the gold standard. This approach was based on a previous study in which nCECT demonstrated superior performance compared to standard-of-care CT angiography (CTA) for detecting preclinical or mild aortopathy before aortic enlargement became evident 14. A comparison of nCECT with non-contrast CT was not performed in this study due to the inherently poor image contrast in differentiating structures, which results in the poor performance of non-contrast CT. A comparison of nCECT with conventional contrast-enhanced CT was also not performed due to the very short blood half-life (~a few minutes) and the long scan time (~10 minutes) associated with acquiring high-resolution CT images on a preclinical micro-CT scanner. The short half-life, combined with the long CT scan acquisition time, leads to poor opacification of the vascular compartment. Furthermore, conventional contrast agents exhibit very rapid wash-in and wash-out kinetics at sites of pathology, resulting in inadequate intramural accumulation and CT signal. These inherent properties of conventional contrast agents preclude their use in preclinical *in vivo* studies for the detection of aortic disease.

Our study has limitations that warrant further investigations. The study showed clearance of intramurally accumulated nanoparticles, as evidenced by gradual return of post-contrast CT attenuation to pre-contrast baseline levels, however, mechanisms governing intramural clearance of NPCA needs to be elucidated and studied further. Mechanisms dictating intramural NPCA distribution patterns needs to be studied further as it could provide clues about propagation patterns for aortic wall injury and dissection eventually leading to aortic rupture. Finally, the influence of mechanisms that drive long term remodeling and recovery process within the degenerated aortic wall and the healing of the aortic endothelium barrier on intramural trafficking of nanoparticles needs to be studied further.

## Conclusion

Nanoparticle contrast-enhanced CT enabled detection and *in vivo* imaging of aortic injury with focally enhanced aortic wall permeability in a mouse model of sporadic AAD. Using all three studied nanoparticle sizes (80, 150, 240 nm), nanoparticle contrast-enhanced CT demonstrated higher sensitivity than gross examination for detecting early stages of aortopathy. A dose dependent improvement in sensitivity was observed with only high NPCA dose (1200 mg I/kg) demonstrating superior performance compared to gross examination. Nanoparticle contrast-enhanced CT enabled *in vivo* interrogation of temporal changes in aortic wall permeability.

## Supplementary Material

Supplementary figures and tables.

## References

[1] Shen YH, LeMaire SA, Webb NR, Cassis LA, Daugherty A, Lu HS (2020). Aortic aneurysms and dissections series. Arterioscler Thromb Vasc Biol.

[2] Shen YH, LeMaire SA, Webb NR, Cassis LA, Daugherty A, Lu HS (2020). Aortic aneurysms and dissections series: Part II: Dynamic signaling responses in aortic aneurysms and dissections. Arterioscler Thromb Vasc Biol.

[3] Wu D, Shen YH, Russell L, Coselli JS, LeMaire SA (2013). Molecular mechanisms of thoracic aortic dissection. J Surg Res.

[4] LeMaire SA, Russell L (2011). Epidemiology of thoracic aortic dissection. Nat Rev Cardiol.

[5] Sakalihasan N, Limet R, Defawe O (2005). Abdominal aortic aneurysm. Lancet.

[6] Richards JMJ, Semple SI, MacGillivray TJ, Gray C, Langrish JP, Williams M (2011). Abdominal aortic aneurysm growth predicted by uptake of ultrasmall superparamagnetic particles of iron oxide: A pilot study. Circ Cardiovasc Imaging.

[7] Saraff K, Babamusta F, Cassis LA, Daugherty A (2003). Aortic dissection precedes formation of aneurysms and atherosclerosis in angiotensin II-infused, apolipoprotein E-deficient mice. Arterioscler Thromb Vasc Biol.

[8] Trachet B, Piersigilli A, Fraga-Silva RA, Aslanidou L, Sordet-Dessimoz J, Astolfo A (2016). Ascending aortic aneurysm in angiotensin II-infused mice: Formation, progression, and the role of focal dissections. Arterioscler Thromb Vasc Biol.

[9] Daugherty A, Powell JT (2014). Recent highlights of *ATVB*: Aneurysms. Arterioscler Thromb Vasc Biol.

[10] Hsu JC, Tang Z, Eremina OE, Sofias AM, Lammers T, Lovell JF (2023). Nanomaterial-based contrast agents. Nat Rev Methods Primers.

[11] Cormode DP, Naha PC, Fayad ZA (2014). Nanoparticle contrast agents for computed tomography: a focus on micelles. Contrast Media Mol Imaging.

[12] Hsu JC, Nieves LM, Betzer O, Sadan T, Noël PB, Popovtzer R (2020). Nanoparticle contrast agents for X-ray imaging applications. Wiley Interdiscip Rev Nanomed Nanobiotechnol.

[13] Devkota L, Zhang C, Bhandari P, Vela D, Starosolski Z, Shen YH (2023). Abstract 271: Interrogation of endothelial permeability in aortic aneurysm and dissection using nanoparticle contrast-enhanced computed tomography. Arterioscler Thromb Vasc Biol.

[14] Ghaghada KB, Ren P, Devkota L, Starosolski Z, Zhang C, Vela D (2021). Early detection of aortic degeneration in a mouse model of sporadic aortic aneurysm and dissection using nanoparticle contrast-enhanced computed tomography. Arterioscler Thromb Vasc Biol.

[15] Ghaghada KB, Badea CT, Karumbaiah L, Fettig N, Bellamkonda RV, Johnson GA (2011). Evaluation of tumor microenvironment in an animal model using a nanoparticle contrast agent in computed tomography imaging. Acad Radiol.

[16] Clark DP, Ghaghada K, Moding EJ, Kirsch DG, Badea CT (2013). *In vivo* characterization of tumor vasculature using iodine and gold nanoparticles and dual energy micro-CT. Phys Med Biol.

[17] Bhavane R, Badea C, Ghaghada KB, Clark D, Vela D, Moturu A (2013). Dual-energy computed tomography imaging of atherosclerotic plaques in a mouse model using a liposomal-iodine nanoparticle contrast agent. Circ Cardiovasc Imaging.

[18] Ghaghada KB, Starosolski ZA, Lakoma A, Kaffes C, Agarwal S, Athreya KK (2016). Heterogeneous Uptake of Nanoparticles in Mouse Models of Pediatric High-Risk Neuroblastoma. PLoS One.

[19] Luo W, Wang Y, Zhang L, Ren P, Zhang C, Li Y (2020). Critical role of cytosolic DNA and its sensing adaptor STING in aortic degeneration, dissection, and rupture. Circulation.

[20] Ren P, Hughes M, Krishnamoorthy S, Zou S, Zhang L, Wu D (2017). Critical Role of ADAMTS-4 in the development of sporadic aortic aneurysm and dissection in mice. Sci Rep.

[21] LeMaire SA, Zhang L, Luo W, Ren P, Azares AR, Wang Y (2018). Effect of ciprofloxacin on susceptibility to aortic dissection and rupture in mice. JAMA Surg.

[22] Ren P, Wu D, Appel R, Zhang L, Zhang C, Luo W (2020). Targeting the NLRP3 inflammasome with inhibitor MCC950 prevents aortic aneurysms and dissections in mice. J Am Heart Assoc.

[23] Ridler TW, Calvard S (1978). Picture thresholding using an iterative selection method. IEEE Trans Syst Man Cybern.

