# Supplementary material for: Nanoparticle contrast-enhanced computed tomography of sporadic aortic aneurysm and dissection: Effect of nanoparticle size and contrast agent dose
Source: Theranostics. 2025 Feb 24;15(8):3462–73. doi: 10.7150/thno.109325 (PMC11905125; doi:10.7150/thno.109325)
Supplement: Supplementary file 1 — Supplementary figures and tables. [file thnov15p3462s1.pdf]

Supplemental Materials:

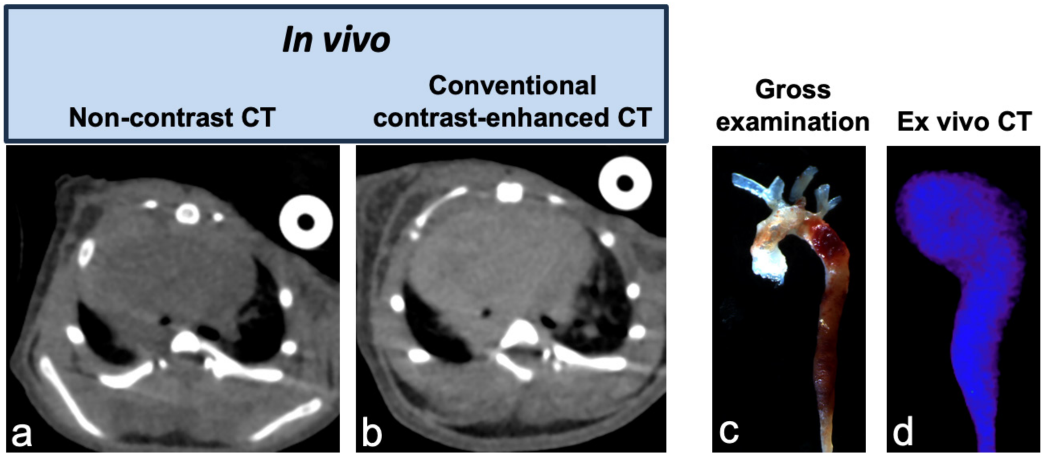

Figure S1. Non-contrast CT and conventional contrast-enhanced CT in a mouse with aortic aneurysm and dissection (AAD). Representative *in vivo* axial (a) non-contrast and (b) conventional contrast-enhanced CT (CECT) images of the thoracic region in a mouse with AAD. At the end of conventional CECT, the mouse was euthanized and aorta harvested for gross examination and *ex vivo* CT. Gross examination (c) shows severe disease in ascending and descending segments of the aorta. However, *in vivo* non-contrast CT and conventional CECT images do not show any evidence of aortic disease. *Ex vivo* CT image also shows no evidence of intramural CT signal at sites of aortic pathology.
